# 

**Published:** 1892-06

**Authors:** 


### CONTENTS:

ORIGINAL ARTICLES: PAGE

Mouse Septicæmia Bacilli in a Pig's Spleen. By Veranus A. Moore, B. S., M. D. 333
Some Parasitic Affections of Animals. By R. R. Dinwiddle.... ................ 342
Hoof Culture. By Williamson Bryden, V. S................................ ... 344
Notes on Parasites. By C. W. Stiles, A. M., Ph. D........................... 346
Actinomycosis in Relation to Meat Inspection. By W. L. Williams, V. S....... 351
Aneurysms in Colic. Translated by C. A. Cary, B. S., D. V. M................ 355
A Consideration of Actinomycosis. By Dr. F. S. Billings..................... 358
Investigation upon the Diseases of Rodents. By S. E. Weber................. 374

EDITORIAL:

Veterinary Inspection at Horse Shows........................................ 385

SELECTION:

The Oath of Hippocrates..................................................... 387

SOCIETY PROCEEDINGS:

Massachusetts Veterinary Association,........................................ 393

Massachusetts Veterinary Association,........................................ 394

Massachusetts Veterinary Association,........................................ 395

